# Long-Range RFID Indoor Positioning System for an Autonomous Wheelchair

**DOI:** 10.3390/s25082542

**Published:** 2025-04-17

**Authors:** João S. Pereira

**Affiliations:** 1 Polytechnic Institute of Leiria, School of Technology and Management, Regional University Network, 2411-901 Leiria, Portugal; joao.pereira@ipleiria.pt; 2 Instituto de Telecomunicações, 2411-901 Leira, Portugal; 3 Centre for Research in Informatics and Communications—CIIC, 2411-901 Leira, Portugal

**Keywords:** RFID, IPS, IIoT, smart wheelchair

## Abstract

A new Radio-Frequency Identification (RFID) indoor positioning system (IPS) has been developed to operate in environments where the Global Positioning System (GPS) is unavailable. Traditional RFID tracking systems, such as anti-theft systems in clothing stores, typically work within close proximity to exit doors. This paper presents a novel RFID IPS capable of locating and tracking passive RFID tags over a larger area with greater precision. These tags, costing approximately EUR 0.10 each, are in the form of small stickers that can be attached to any item requiring tracking. The proposed system is designed for an autonomous wheelchair, built from scratch, which will be identified and monitored using passive RFID tags. Our new RFID IPS, with a 12 m range, is implemented in this “smart” wheelchair.

## 1. Introduction

The Industrial Internet of Things (IIoT) holds significant potential for the business sector by integrating machines, advanced analytics, and human oversight. This technology functions as a sensory network of electronic devices that monitor industrial processes, collect and exchange data, and continuously optimize manufacturing operations. Passive Radio-Frequency Identification (RFID) tags are widely recognized as key enablers of the IIoT, offering a convenient and wireless identification solution, typically used in short-range applications.

The Global Positioning System (GPS) is the most widely used technology for object tracking; however, its effectiveness is limited in enclosed spaces, and it has a positioning accuracy of approximately 5 m. To overcome this limitation in indoor environments, a long-range RFID-based solution has been developed. This approach utilizes a reader connected to a directional antenna and multiple low-cost passive RFID tags affixed to various points to estimate the position of a wheelchair within a large indoor space.

Indoor positioning systems (IPSs) are a well-established and extensively researched topic in both the scientific literature and commercial applications. These systems enable a wide range of innovative object-tracking solutions using electronic methods. One key application is the identification of tools, industrial materials, and manufactured goods within warehouses and industrial spaces. Despite the abundance of existing IPS solutions, research in this field remains active worldwide. For instance, the Instituto de Telecomunicações and the Polytechnic Institute of Leiria are investigating indoor positioning through projects that leverage radio-frequency technology [1,2,3].

Numerous companies worldwide are developing IPS solutions, as referenced in [4,5,6]. One of the most widely advertised wireless solutions is Pozyx, which achieves an estimated positioning error of approximately 10 centimeters in an average-sized room. Additionally, various national and international companies offer commercial IPS solutions. For example, RCSoft (Coimbra, Portugal) [7] provides IPS technology, while the View Technologies consortium—established in 2014 as a joint venture between Stanley Black & Decker, Inc. (New Britain, CT, USA) and RF Controls—holds an extensive portfolio of localization patents in the global market [8].

Many national and international RFID tracking solutions have not been adequately adapted to the challenges of industrial environments, where electromagnetic interference from electrical machinery is predominant. Multipath interference is a key factor limiting the long-range readability of RFID tags [9]. In the mold industry, where metallic surfaces reflect electromagnetic waves, multipath interference presents the greatest obstacle to implementing a reliable long-range RFID positioning system with passive tags [10,11].

This paper presents an innovative radio-frequency positioning system designed to overcome the limitations of existing IPS solutions in industrial and hospital environments. The proposed system employs passive anti-metal RFID tags (ISO 18000-6C) [12] and a long-range IPS framework incorporating Ultra-High-Frequency (UHF) RFID readers and directional antennas. To ensure patent compliance, our solution is based on patents from the Polytechnic Institute of Leiria and the Institute of Telecommunications, avoiding infringement on View Technologies’ intellectual property [13,14,15].

To simplify the IPS, we propose a mathematical model based on the Received Signal Strength Indicator (RSSI), enabling tag positioning by analyzing the intersection of radiation patterns from two RFID readers. While other localization systems using different techniques [16,17] achieve similar accuracy, they require a minimum spacing of 0.50 m between tags. In contrast, our IPS can further enhance accuracy by incorporating multiple pairs of RFID readers.

While it is true that RSSI-based positioning alone presents challenges due to signal attenuation, multipath effects, and interference, our approach focuses on leveraging long-range RFID in a novel configuration to maximize its applicability in large-scale environments. Unlike traditional RFID implementations, our system is designed to operate efficiently over extended distances with minimal infrastructure, providing a practical and cost-effective alternative to more complex solutions, such as Ultra-Wideband (UWB) or computer vision-based systems. Although UWB systems offer superior accuracy, their cost and implementation complexity often limit their feasibility in real-world deployments, especially for large-scale indoor environments. Our goal is not to compete with state-of-the-art machine-learning-enhanced positioning but, rather, to provide a viable and scalable alternative that maintains accuracy while ensuring ease of deployment and affordability [18].

We acknowledge that RFID-based positioning has been widely explored; however, our system differentiates itself by focusing on long-range passive RFID tracking without requiring significant infrastructure investments. Unlike some existing RFID positioning systems that rely on complex triangulation or fingerprinting methods, our approach is optimized for deployment in environments where cost and scalability are primary concerns. Many IPS solutions that integrate hybrid techniques—such as RFID combined with cameras or UWB—require additional hardware, computational resources, and maintenance, which may not always be practical. To further improve accuracy, we plan to explore enhancements such as dynamic RSSI calibration and environmental adaptation models to mitigate common limitations associated with RSSI-based tracking [19].

Incorporating sensor fusion techniques can significantly enhance positioning accuracy, and we are investigating ways to integrate additional sensors, such as Inertial Measurement Units (IMUs), accelerometers, and LiDAR, into our system if the cost is not too high. A promising approach is the use of complementary filtering or Kalman filters to refine position estimates by combining RFID data with motion-tracking information. Sensor fusion techniques have been shown to improve positioning accuracy in dynamic environments, and future iterations of our system will include probabilistic localization models that account for environmental variability. Our objective is to strike a balance between cost-effective deployment and improved accuracy by leveraging lightweight sensor fusion methods [20].

The RSSI curve intersection method currently employed in our system is indeed a fundamental approach to localization; however, we are exploring more advanced techniques to improve real-world applicability. Future developments may incorporate adaptive signal strength models that account for environmental factors such as obstacles, multipath interference, and signal decay. One potential enhancement involves implementing machine-learning-based regression models trained on environmental data to dynamically adjust the RSSI values based on known interference patterns. Additionally, implementing probabilistic models, such as Gaussian processes or Bayesian inference, could further refine the location estimates in complex indoor environments [21].

Multipath interference is a well-known challenge in RFID-based positioning, and we recognize the need for robust compensation mechanisms. To address this, we are evaluating the use of particle filtering and k-Nearest Neighbor (kNN) fingerprinting methods to improve accuracy in noisy environments. By creating an adaptive localization model that incorporates historical RSSI data, we can mitigate the impact of environmental variability. Additionally, ongoing research suggests that deep learning techniques, such as convolutional neural networks (CNNs), could be trained on spatial RSSI distributions to enhance positioning accuracy in multipath-prone settings. These improvements will be explored in future work to optimize the robustness of our system [22].

Our current implementation using two RFID readers and passive tags represents a baseline prototype; however, we acknowledge that scalability is crucial for broader applications. Expanding the system to include multiple readers in a distributed configuration will significantly enhance its coverage and accuracy. Furthermore, hardware modifications, such as customized RFID antennas with optimized beamforming techniques, can improve signal consistency and positioning precision. We have a pending patent for this solution. We are also investigating the potential integration of hybrid RFID–Wi-Fi tracking to enhance location accuracy while maintaining the advantages of passive RFID tracking. Future versions of our system will incorporate these improvements to increase its scalability and competitiveness with other IPS solutions.

The application of our RFID-based tracking system for wheelchair navigation serves as a proof of concept, demonstrating the potential for low-cost, infrastructure-light mobility assistance. While existing technologies such as LiDAR-based navigation and SLAM (Simultaneous Localization and Mapping) offer high accuracy, they often require expensive hardware, extensive calibration, and substantial computational resources. Our approach aims to provide a complementary alternative that leverages RFID’s ability to track objects and guide mobility devices with minimal overhead. Additionally, we are exploring ways to integrate RFID-based navigation with existing mobility assistance technologies to enhance their usability and accessibility.

This paper introduces a novel RFID-based IPS optimized for autonomous wheelchair navigation in indoor environments. Unlike traditional GPS tracking, which is limited to outdoor spaces, this solution leverages passive RFID tags and a refined RSSI-based mathematical model to estimate position. The main technical contributions of this work include the following:

1. A refined RFID-based localization model that improves accuracy by incorporating an RSSI-based surface model and curve intersection techniques for enhanced tag position estimation.
2. A comprehensive performance evaluation, including error analysis, real-time tracking speed, and experimental validation in both small-scale and large-scale environments.
3. A prototype implementation integrated into an autonomous wheelchair, demonstrating the applicability of RFID tracking in assistive mobility solutions.

By systematically addressing accuracy limitations in traditional RSSI-based approaches, this system offers a low-cost and scalable alternative to UWB-, LiDAR-, and vision-based positioning.

The remainder of this paper is structured as follows:

Section 2 reviews RFID-based indoor positioning systems, highlighting their advantages, challenges, and existing solutions. This section also presents a comparative analysis of IPS technologies, positioning algorithms, and their applicability to mobility assistance.

Section 3 describes the system architecture, detailing the hardware and software components used in the RFID tracking system, along with an overall schematic of the setup.

Section 4 presents the first phase of the project, outlining the software implementation, experimental setup, and initial RSSI-based reading tests used to validate the system’s feasibility.

Section 5 introduces the RSS-based mathematical model for localization, explaining the key equations, intersection methods for tag position estimation, and error analysis.

Section 6 discusses the application of the system in an autonomous wheelchair, detailing integration challenges, real-world testing, and performance evaluation.

Section 7 analyses the experimental results, compares different positioning methods, and assesses the system accuracy and error margins. A discussion on system scalability and deployment challenges is also included.

Section 8 concludes this paper, summarizing the key findings and contributions. Future work will focus on integrating AoA-based tracking, improving computational efficiency, and evaluating the system in larger real-world environments.

This structured approach provides a comprehensive analysis of the design, implementation, and performance evaluation of the proposed RFID-based positioning system.

## 2. RFID Technology

### 2.1. Review Stage

RFID technology belongs to the Automatic Identification and Data Capture (AIDC) category, which encompasses various technologies that enable machines to identify objects autonomously [23]. These technologies facilitate automated data capture, allowing companies to track objects without human intervention. An RFID tag attached to an object transmits data, which are automatically identified, stored, and analyzed on a computer. The following sections provide a brief overview of existing IPSs.

The origins of RFID technology date back to the early 20th century, with the advent of radio-wave communication and radar development [24]. Its first practical application emerged during World War II, when Allied forces used RFID for Friend-or-Foe (FoF) identification, a system designed to distinguish friendly aircraft from enemy aircraft [25]. This system utilized a transponder attached to the aircraft, which responded to radar signals, allowing it to be identified as friendly and reducing the risk of friendly fire incidents.

In the 1960s, RFID technology became commercially available as an anti-theft measure under the name Electronic Article Surveillance (EAS). The system utilized 1-bit tags to detect the presence or absence of a tag, providing an effective and cost-efficient method for preventing shoplifting—a practice that is still widely used today.

In the 1980s, RFID technology saw widespread implementation, with notable developments in both the United States and Europe [26]. In the USA, research primarily focused on transportation, personnel access, and, to a lesser extent, animal tracking. In Europe, the emphasis shifted toward animal tagging, industrial and commercial applications, and electronic toll collection [27].

An RFID system consists of three primary components: the antenna, reader, and transponder (RFID tag). The antenna, connected to the reader, can be customized to suit the specific requirements of the project and its application. Similarly, the RFID tag features a small antenna, typically designed to match the system’s operating frequency.

**Figure 1:**
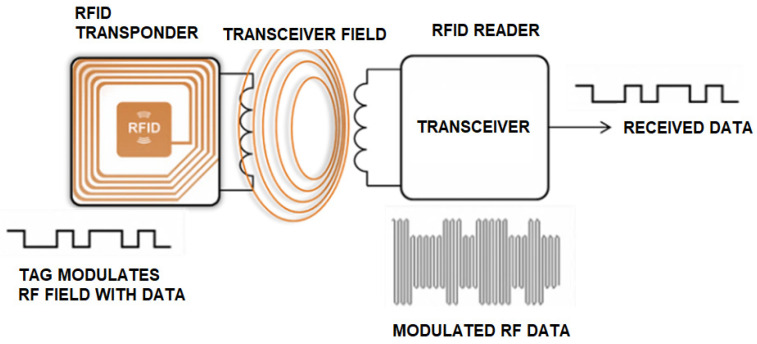
RFID architecture.

When the reader/writer antenna transmits a signal, it activates the tag’s interface, allowing the stored data to be read or written. This process is illustrated in Figure 1 [28]. Antennas vary in size and shape based on factors such as frequency, range, and directionality, making them adaptable to a wide range of applications. The RFID reader can operate continuously in scenarios requiring the reading of numerous tags, such as toll collection systems, or it can be activated and deactivated as needed [29].

**Figure 2:**
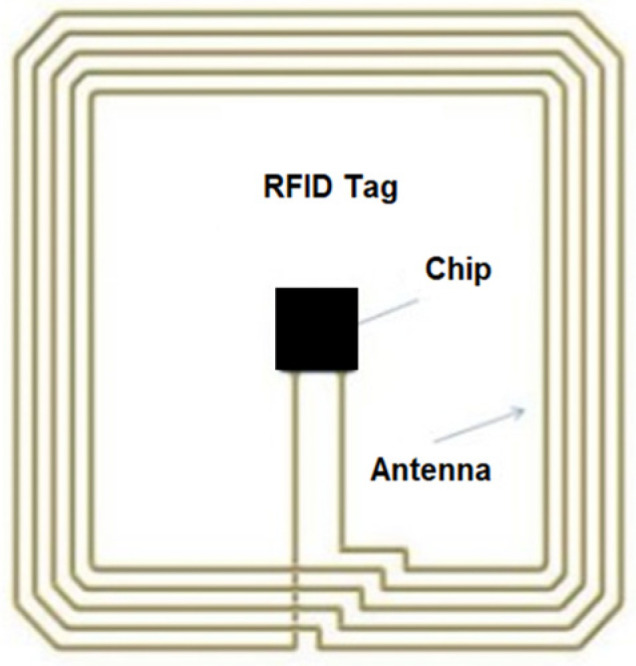
RFID tag.

In this document, the data transport device, referred to as an RFID tag, consists of an electronic chip and a coupling element, as shown in Figure 2 [30]. Passive tags do not have their own power source, and they rely on the reader to send, receive, and/or modify data through the tag’s antenna. These tags are available in various sizes and shapes, making them suitable for a wide range of applications.

### 2.2. Different Types of Tags

RFID tags can be divided into three major types: active, passive, and semi-passive.

Active tags are equipped with their own power source, typically a battery, and operate using UHF, ranging from 300 MHz to 3 GHz. These tags offer a longer range, with some capable of transmitting data up to 100 m. Due to their larger size and higher cost, they are primarily used to track valuable assets. Active tags often include sensors that measure various parameters, such as temperature, humidity, and light. These tags are categorized into two types: transponders and beacons. Transponders become active and transmit data when they receive a signal from a reader’s antenna. An example of this type is used in electronic toll payment systems, where the tag remains dormant until the user passes through the toll booth. Beacons, on the other hand, emit signals at predefined intervals and are commonly used in Real-Time Location Systems (RTLSs) to track cargo containers or wheelchairs in hospitals. While these tags consume battery power, they are generally much more expensive than passive tags, often costing up to 100 times more.

**Figure 3:**
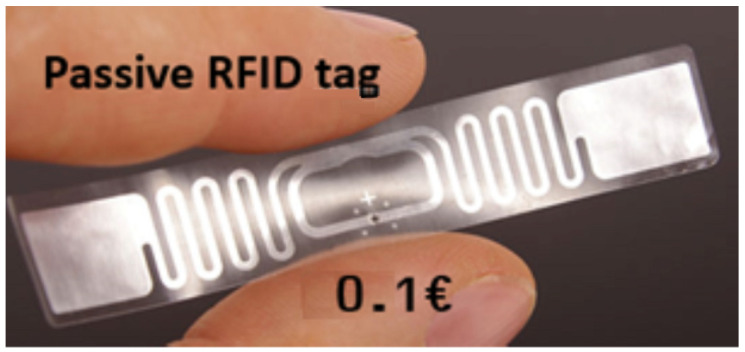
Passive RFID tag.

Passive RFID tags rely on energy harvested from the reader’s signal to transmit data back to the reader, as they do not have an internal power source. These tags can operate using LF (Low Frequency, 30 to 300 kHz), HF (High Frequency, 3 to 30 MHz), or UHF (Ultra-High Frequency, 300 to 3000 MHz), although their reading range is typically shorter due to the limited power available. Passive tags are generally smaller, more affordable, and more versatile than active tags, making them suitable for tracking a wide range of objects. A depiction of a passive RFID tag is shown in Figure 3.

Semi-passive tags combine the characteristics of both active and passive tags. They are equipped with an internal power source, which allows them to use the energy received from the reader more efficiently, thereby improving data transfer rates and read distances. RFID systems come in various implementations and categories, and tags can be classified as either read-only or read/write. Read-only tags are pre-programmed with a fixed set of data, typically ranging from 32 to 127 bits, which cannot be altered. On the other hand, read/write tags can store different information over time, allowing for greater flexibility. RFID tags are categorized into five classes: Class 0: passive, read-only; Class 1: passive, write once, read many; Class 2: passive, multi-write and -read; Class 3: active, multi-write and -read; Class 4: active, networking tags.

### 2.3. Frequency Bands

RFID technology operates across various frequencies within the electromagnetic spectrum, specifically in the Industrial, Scientific, and Medical (ISM) bands, which are exempt from licensing requirements. These same frequency bands are also utilized by other wireless technologies, such as Wi-Fi and Bluetooth. To illustrate the frequencies used in different RFID applications, along with the associated benefits and drawbacks of operating within these bands, Table 1 outlines some commonly used RFID tag types and their respective applications.

### 2.4. Standards

RFID tags can be identified using various standards based on their intended application. Some tags may even use proprietary identification systems that do not adhere to any established standard. However, the most widely adopted identification standard for UHF RFID tags is the Electronic Product Code (EPC), which was introduced by the Auto-ID Centre. The EPC has two variations: a 64-bit code and a 96-bit code. The 96-bit code provides unique identifiers for up to 268 million companies, offering 16 million different object classes, each with 68 million serial numbers [30]. In contrast, the 64-bit code is a more cost-effective alternative, although it supports fewer serial numbers.

**Figure 4:**
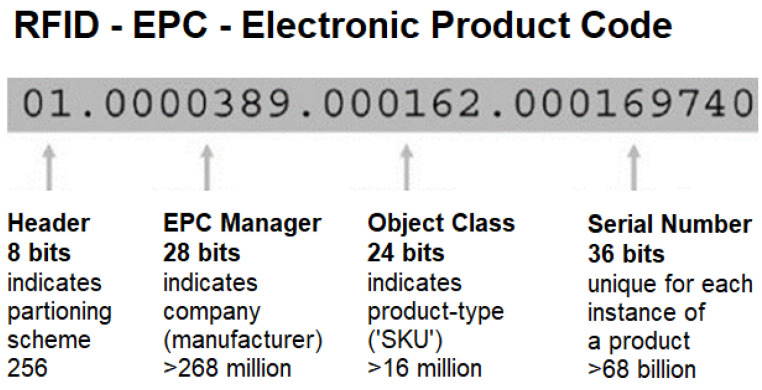
Tag EPC.

The EPC consists of a header and three distinct data sections, as illustrated in Figure 4 [31]. The header indicates the version of the EPC in use, the second section identifies the manufacturer’s code, and the third part specifies the product type, typically the Stock=Keeping Unit (SKU) [32]. A key distinction between the EPC and traditional barcodes is the inclusion of a unique serial number.

### 2.5. RFID in Localization

Indoor environments present significant challenges for radio waves’ propagation, such as multipath interference, line-of-sight (LOS) limitations, absorption, diffraction, and reflection. These issues can lead to inaccuracies in signal readings and errors in tag localization. To overcome these challenges, several location algorithms have been developed, which can be categorized into three primary types:

- Distance estimation;
- Scene analysis;
- Proximity.

### 2.6. Distance Estimation Algorithms

Distance estimation algorithms utilize the triangulation method to determine the location of a target. This approach involves measuring the Angle of Arrival (AoA) from at least two reference points, which allows for the determination of the target’s position at the point of convergence [33,34,35,36,37,38,39,40]. The estimated position is then obtained by the intersection of the lines defined by these angles.

There are other techniques available for locating objects, such as Received Signal Strength (RSS), Time of Arrival (TOA), Time Difference of Arrival (TDOA), and Received Signal Phase (RSP).

RSS—This technique estimates the location of a tag by measuring the attenuation of the signal power transmitted between the transmitter and receiver.

TOA—Time of Arrival is another technique used for object localization, where the time taken for the signal to travel from the tag to the receiver is measured, and the distance between the tag and the reference point is directly proportional to the propagation time of the signal.

TDOA—This algorithm determines the location of a tag by calculating the time difference at which the signal emitted by the tag reaches multiple measuring units. By comparing the time differences between two or more receivers, the tag’s relative position can be estimated.

RSP—Also known as POA (Place of Arrival), this algorithm estimates the distance by using the signal’s phase delay, which is expressed as a fraction of the signal’s wavelength. Specific sinusoidal signals must be emitted from known locations.

### 2.7. Scene Analysis Algorithms

Scene analysis algorithms generally involve two key steps: first, collecting environmental data using anchor tags strategically placed in key locations (often referred to as fingerprints); and second, estimating the tag’s localization by comparing the collected measurements with the pre-established set of fingerprints. The two primary algorithms employed in this approach are kNN (k-Nearest Neighbor) [41] and various probabilistic methods.

**Figure 5:**
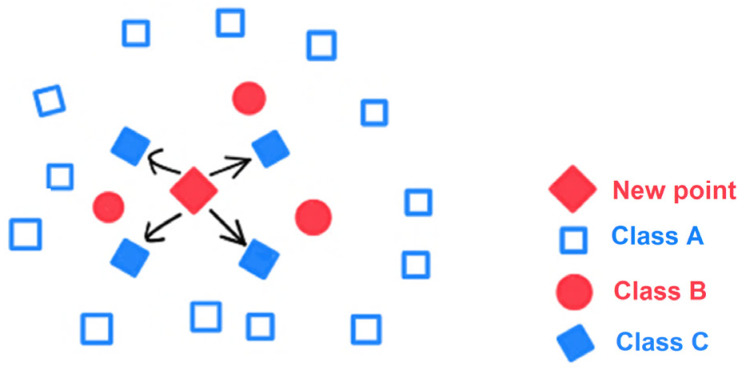
kNN technique.

The algorithms can be described as follows:

kNN (k-Nearest Neighbor)—This method begins by measuring the RSS values of tags at known locations to create a database of RSS data, often referred to as a radio map. When a new measurement is obtained from a reader, the k nearest matches in the radio map are identified and used to estimate the tag’s location. This is typically carried out using the root-mean-square error principle, as shown in Figure 5.

**Figure 6:**
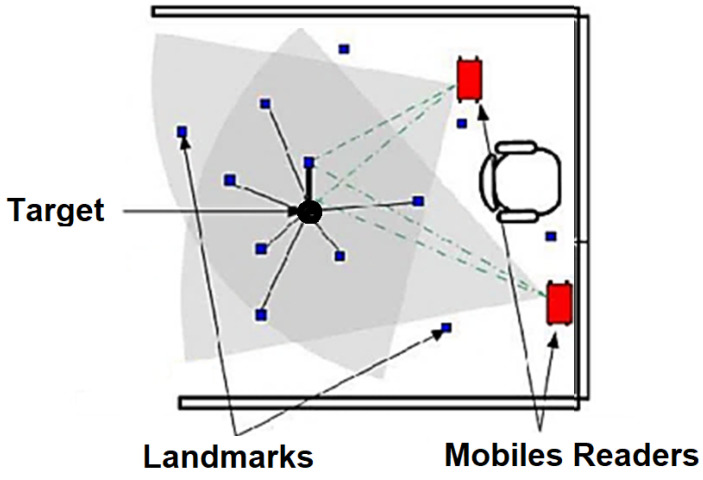
Probabilistic location.

Probabilistic approach—This method estimates the tag’s location by considering a set of n possible locations and a single observed signal strength vector, as illustrated in Figure 6 [18]. The location with the highest probability is then selected. To improve the localization accuracy, these approaches usually involve techniques like calibration, active learning, error estimation, and historical tracking [42].

**Figure 7:**
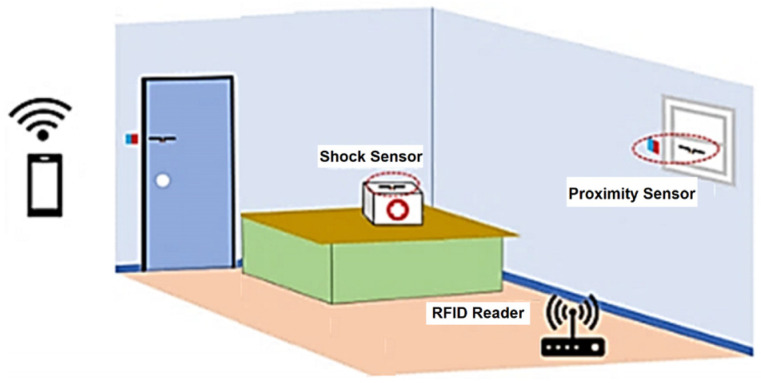
Proximity technique.

Proximity algorithms—As the name suggests, proximity algorithms rely on the proximity of the tag to the reader antenna, as shown in Figure 7 [43]. This method is primarily based on signal strength. When a tag enters the range of a single antenna, its location is considered to be the same as the receiver’s location. If one or more antennas detect the tag, it is assumed to be within the range of the antenna with the highest signal strength. While this is the simplest method to implement, it is also the least accurate.

The review of RFID-based indoor positioning systems in this section highlights both the advantages and limitations of existing approaches. While RFID technology provides a cost-effective and scalable alternative to the GPS for indoor environments, traditional RSS-based positioning faces challenges related to multipath interference, signal attenuation, and environmental variability. Several improvements, such as fingerprinting, Time of Arrival (ToA), and hybrid sensor fusion techniques, have been proposed in the literature to enhance accuracy, but they often require complex infrastructure, increased computational costs, or additional hardware.

Given these insights, the proposed system aims to optimize an RFID-based IPS using a mathematical model that refines RSS readings to improve localization accuracy while maintaining low-cost deployment and real-time processing capabilities. The following section presents the system architecture, detailing the hardware and software components used in the implementation of our RFID-based localization approach.

## 3. Technologies Used

This chapter provides an overview of the hardware and software used in the development of this project. It begins by detailing the hardware evaluated for precise object identification, data processing, and position calculation.

### 3.1. Hardware Used

**Figure 8:**
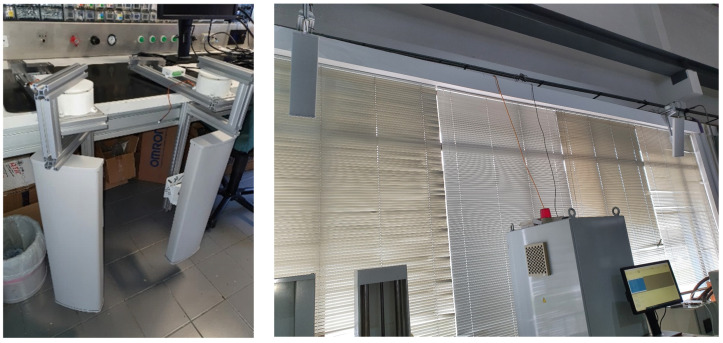
RFID antennas over rotating bases.

To set up the RFID system, two readers, two antennas, and several UHF passive tags were required. Additionally, two ESP8266 microcontrollers with Wi-Fi capabilities were used to collect data from the readers, process them, and calculate the positions of the tags. The antennas were mounted on rotating bases, as shown in Figure 8, allowing movement for enhanced accuracy. However, during the initial testing phase, two static antennas without rotation were used.

### 3.2. RFID Reader

**Figure 9:**
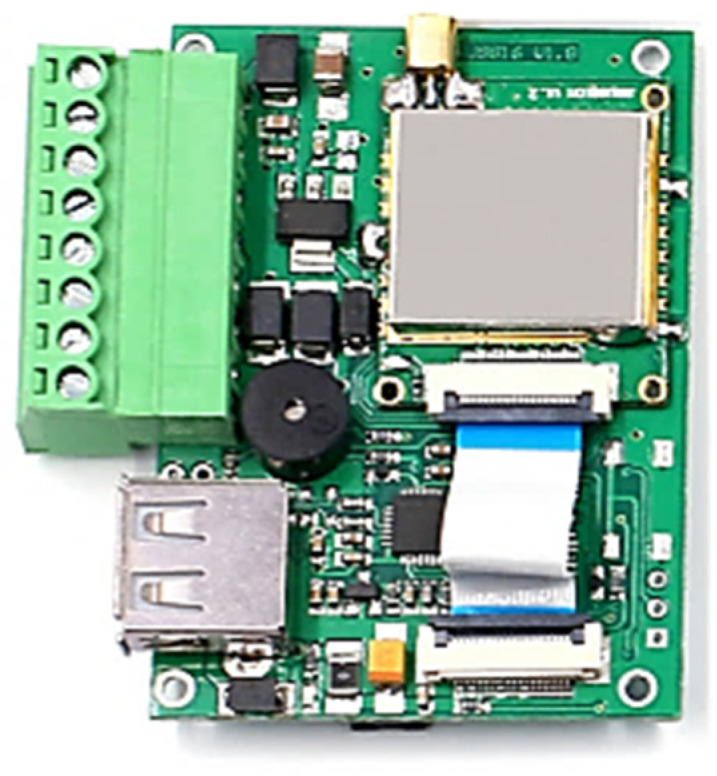
RFID reader (CF-MU904).

The RFID system utilizes a reader module for tag detection, which can be connected to antennas with varying ranges, depending on the application requirements. In this project, a long-range antenna was paired with a reader from Chafon (Shenzhen, China), a company specializing in RFID technology. Figure 9 illustrates the MU904 (ISO 18000-6C) model [44], which operates within the UHF band of 865–868 MHz (EU) or 902–928 MHz (US), adhering to standard RFID frequencies. The reader can process up to 50 tags per second and offers a reading range of 0 to nearly 12 m, depending on the antenna configuration. It can be connected to a device via USB or serial communication (RS232), and Chafon provides a Software Development Kit (SDK) and demo software for seamless integration with the reader.

### 3.3. UHF 12 dBi Antenna (CF-RA1202)

**Figure 10:**
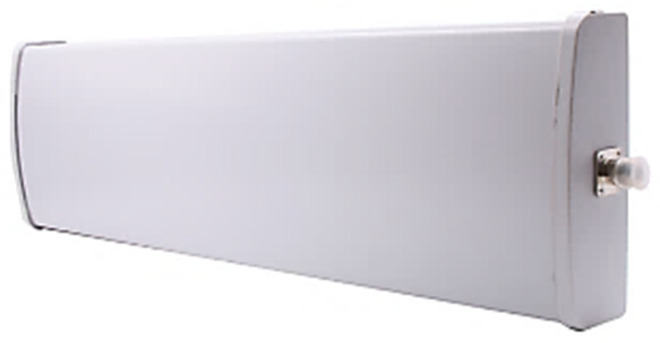
UHF 12 dBi Antenna (CF-RA1202).

**Figure 11:**
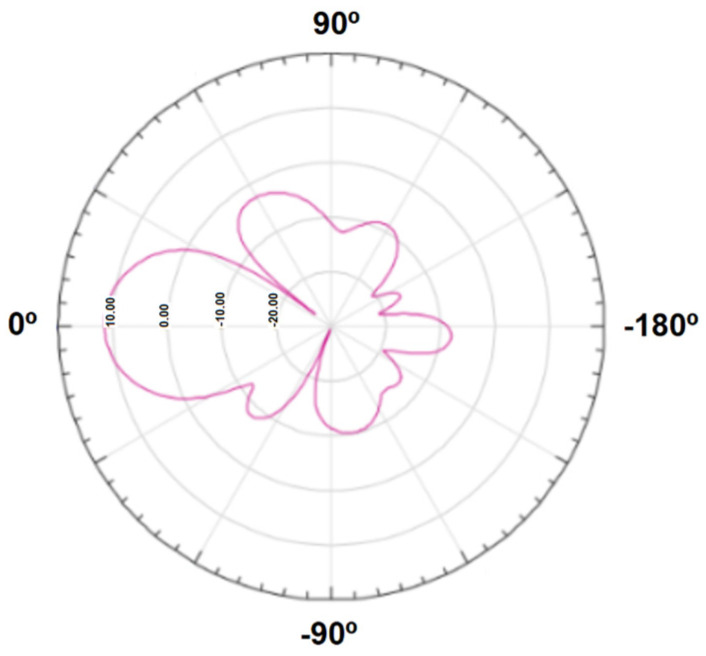
Theoretical radiation diagram of the antenna [dBi].

To extend the reader’s signal and facilitate tag reading, an antenna is essential. In this project, the CF-RA1202 12 dBi antenna from Chafon [45] was used to enhance the reader’s range and effectively detect tags, as shown in Figure 10. The antenna operates within the 860–960 MHz frequency range, aligning with our reading standards, and boasts a gain of 12 dBi. Its durable fiberglass construction ensures robust protection against various environmental conditions, making it ideal for applications such as toll payments on highways. Figure 11 shows the theoretical radiation pattern of the antenna.

### 3.4. Tags

**Figure 12:**
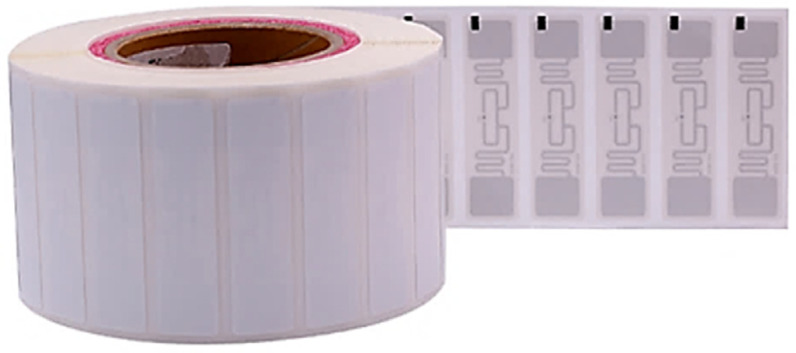
RFID tags used.

In this project, Chafon RFID tags, produced by the same company as the reader and antenna, were utilized, as shown in Figure 12. These tags operate within the UHF radio spectrum (860–960 MHz), matching the frequency range of the antenna. They are passive tags and, when paired with the 12 dBi long-range antenna, they can be read at distances of up to 15 m. The tags feature a compact design measuring 100 × 10 mm and are supplied in rolls with adhesive backing for convenient attachment to objects.

### 3.5. Software Used

This project incorporated various software components, including the .NET Framework for managing the RFID reader, a MySQL database for locally storing the data of read tags, Chafon’s SDK [46] for seamless integration, and a web server to display the real-time locations of passive RFID tags attached to objects.

## 4. First Phase of the Project

This section provides a chronological overview of the project’s development, from the initial concept to the implementation of the first functional prototype. The primary focus was to design a cost-effective, long-range, RFID-based indoor positioning system, using commercially available hardware and software solutions. The development process involved selecting appropriate technologies, configuring the RFID reader, conducting controlled reading tests, and refining the localization model based on experimental results.

### 4.1. Software and System Integration

A key requirement for this project was to ensure the use of low-cost hardware and technologies while maintaining reliability and scalability. To achieve this, the Chafon SDK [46], compatible with the .NET Framework, was selected as the primary software interface for the RFID system. This SDK provided built-in communication protocols that allowed for seamless integration with the RFID reader. Additionally, the ESP8266 microcontroller was employed to facilitate wireless data transmission via Wi-Fi.

**Figure 13:**
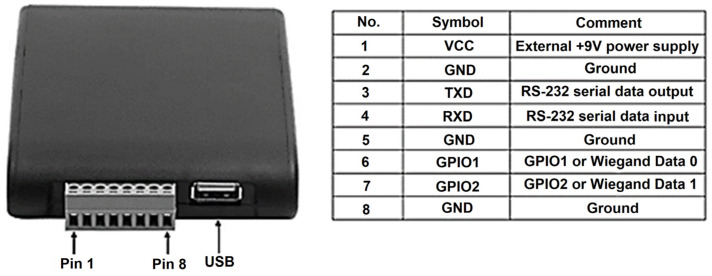
Reader’s connection pins.

The RFID reader module, as illustrated in Figure 13, included two communication interfaces: USB and serial (RS232), accessible through pins 3 and 4. The ESP8266 microcontroller was programmed to read data from the RFID module via the serial port and then transmit the collected data in real time to an external cloud-based server hosted at https://deep-ai-plus.com (accessed on 14 April 2025). This architecture enabled remote access to the collected RFID data, supporting further analysis and system optimization.

To improve data reliability and transmission efficiency, a buffering mechanism was implemented within the microcontroller to prevent packet loss during Wi-Fi communication. Additionally, error detection techniques were applied to filter out inconsistent readings due to environmental interference. These software-based enhancements contributed to stabilizing the system’s performance and ensuring more accurate positioning estimates.

### 4.2. RFID Reading Tests and Performance Evaluation

The first experimental phase aimed to evaluate the reading range and accuracy of the RFID system under controlled conditions. The Chafon RFID reader and antenna were tested in various configurations to analyze the dBm variations in the Received Signal Strength Indicator (RSSI) with respect to distance and orientation. The primary objectives of these tests were as follows:

(1) To determine the actual range of passive RFID tags under ideal conditions.
(2) To assess the impact of tag orientation and polarization on the reading performance.
(3) To validate the radiation pattern of the antenna compared to the manufacturer’s specifications.

For these tests, passive RFID tags were placed on a plastic table at a height of 1 m to minimize ground interference. The initial results showed that the maximum achievable range was 12 m, albeit with low accuracy beyond 8 m due to increased RSSI fluctuations. A critical observation was that the tag’s alignment with the antenna’s polarization significantly affected the readability—when the antenna was horizontally polarized, the tag needed to be aligned horizontally for optimal performance.

To assess the system’s scalability, additional factors were considered:

▪ Interference sources: Wi-Fi networks, metallic objects, and ambient noise.
▪ Tag density: The system successfully detected up to 30 passive tags simultaneously.

**Figure 14:**
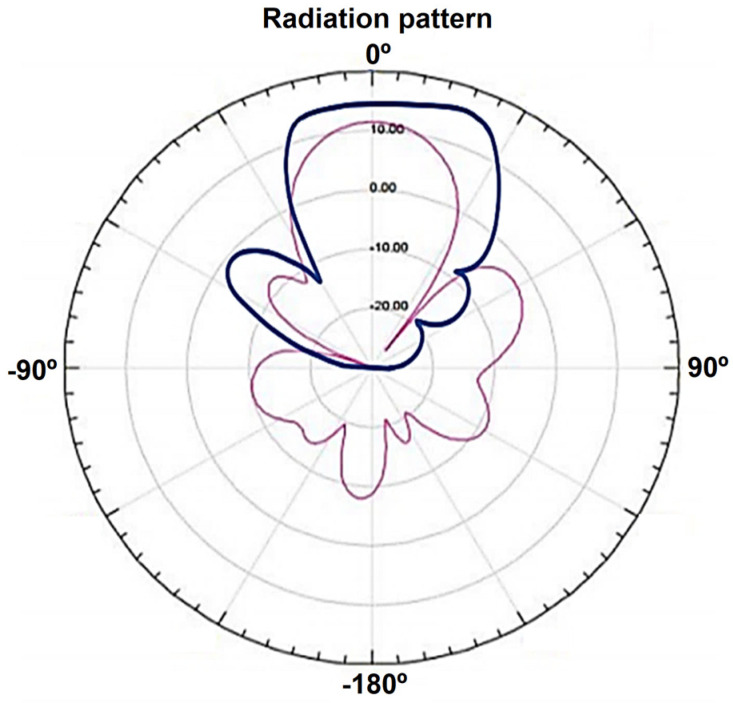
Chafon’s antenna and testing overlay [dBi].

Figure 14 presents a comparative analysis of the Chafon antenna’s theoretical radiation pattern (pink curve) versus the measured results from our experiments (blue curve). These findings confirmed the expected directional behavior of the antenna and highlighted the limitations of using RSSI alone for accurate positioning.

In conclusion, a new mathematical model was developed and implemented to enhance the performance of the RFID-based localization system using the RSSI method for a specific antenna. This model aimed to improve the positioning accuracy by refining the signal strength measurements, compensating for environmental interference, and optimizing the data processing techniques. By systematically analyzing the RSSI variations, the model allowed for more precise estimations of tag locations within the designated tracking area. The results demonstrate the feasibility of using low-cost RFID technology for indoor localization, laying the foundation for further improvements, such as integrating Angle-of-Arrival techniques to enhance accuracy and system robustness.

## 5. The RSS Mathematical Model

In this section, we derive the equations governing the estimation of the tag’s position based on RSSI values from multiple RFID anchors.

### 5.1. RSSI Surface

The RSSI at a given receiver follows the log-distance path loss model, which is expressed as follows:

(1) $$P_{r}=P_{t}-10n{log}_{10}\left(\frac{d}{d_{0}}\right)+X_{\sigma }$$

where

$P_{r}$ is the received power (in dBm);

$P_{t}$ is the transmitted power (in dBm);

*n* is the path loss exponent (which depends on the environment);

*d* is the distance between the transmitter (tag) and receiver (anchor);

$d_{0}$ is a reference distance (usually 1 m);

$X_{\sigma }$ is a Gaussian random variable representing shadowing effects (mean 0, standard deviation σ).

Rearranging this equation to solve for distance *d*, we obtain the following:

$$d=d_{0}*10^{\frac{P_{t}-P_{r}}{10n}}$$

which allows us to estimate the distance from the tag to each anchor based on measured RSSI values.

Given that the position of the tag (*x*, *y*) is unknown, but the positions of the RFID anchors are known, we can express the distances computed from the RSSI measurements as follows:

(2) $${\left(x-x_{i}\right)}^{2}+{\left(y-y_{i}\right)}^{2}=d_{i}^{2}\;\;\;\forall i\in \{1,\;2,\;\ldots ,\;N\}$$

where

(*x*, *y*) are the unknown coordinates of the tag;

(*x_i_*, *y_i_*) are the known coordinates of the *i*-th anchor;

*d_i_* is the estimated distance from the tag to the *i*-th anchor, computed from Equation (1).

This forms a system of nonlinear equations that must be solved to determine *x* and *y*.

The position of the tags can be determined using a mathematical model that finds the intersection between the two RSSI curves of the antennas [47]. Equation (3) was experimentally derived based on the RSSI (*z*) readings along the range *y* = *d* of Equation (1), for two types of RFID antennas:

(3) $$y=\frac{L}{\alpha -\beta }\sqrt{{\left(\alpha -z\right)}^{2}-{\gamma^{2}\left(x-x_{0}\right)}^{2}}$$

where α represents the maximum measured RSSI at the reference (*x*_0_, 0), and β denotes the RSSI at the boundary position (*x*_0_, *L*). The parameter γ is an experimentally determined constant that depends on the antenna characteristics (set to 20 for long-range antennas in this study).

**Figure 15:**
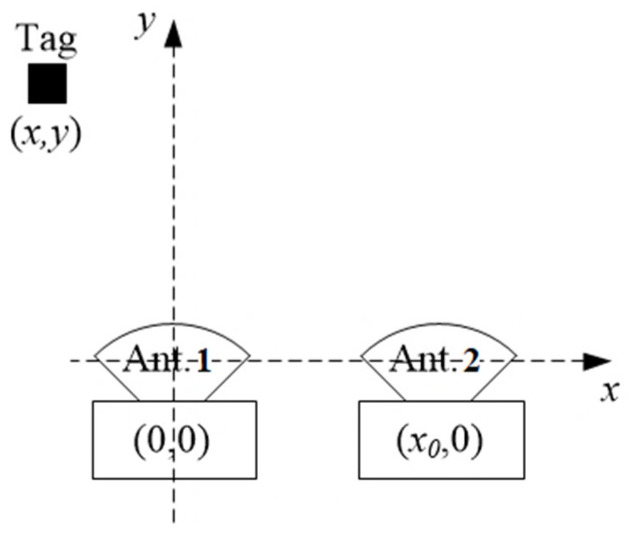
Representation of the IPS space when the tag and 2 RFID readers are within a 5 × 5 m area.

Table 2 lists the variables used in (3) when two RFID directional antennas are positioned at the Cartesian coordinates (0, 0) and (*x*_0_, 0) and a tag is located at position (*x*, *y*), as shown in Figure 15. This figure is the representation of the IPS space when the passive tag and two RFID readers are within a 5 × 5 m area and the two readers are located at the edge of the square (the tag range is 5 m), with the two antennas and the tag placed at a height of 1 m. The passive RFID tag is affixed to the wheelchair, ensuring alignment with antenna polarization, and the system uses two fixed readers for RSSI-based tracking. We assume a free-space indoor environment with no obstacles.

Solving (3), the RSSI sensitivity *z* is given by the following equation:

(4) $$z=\alpha -\sqrt{{\left(\frac{\alpha -\beta }{L}\right)}^{2}y^{2}-{\gamma^{2}\left(x-x_{0}\right)}^{2}}.$$

**Figure 16:**
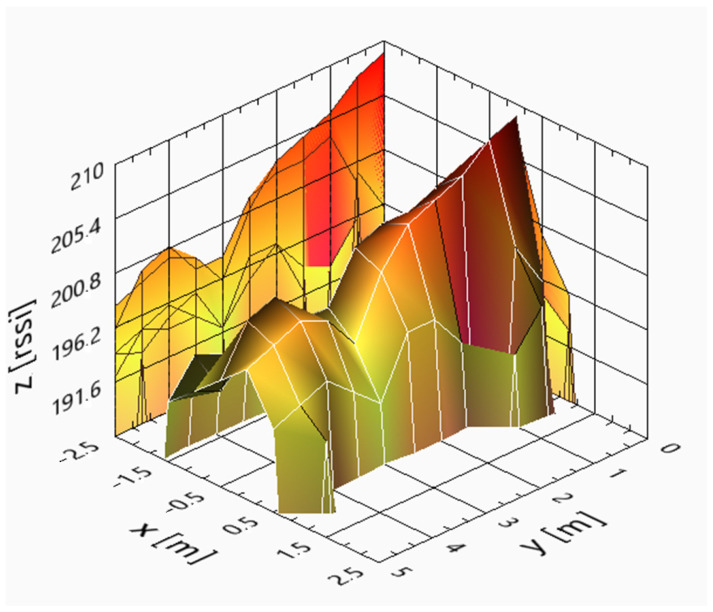
Real RSSI [dBm] surface of the CF-RA1202 antenna when the area is 5 × 5 m.

**Figure 17:**
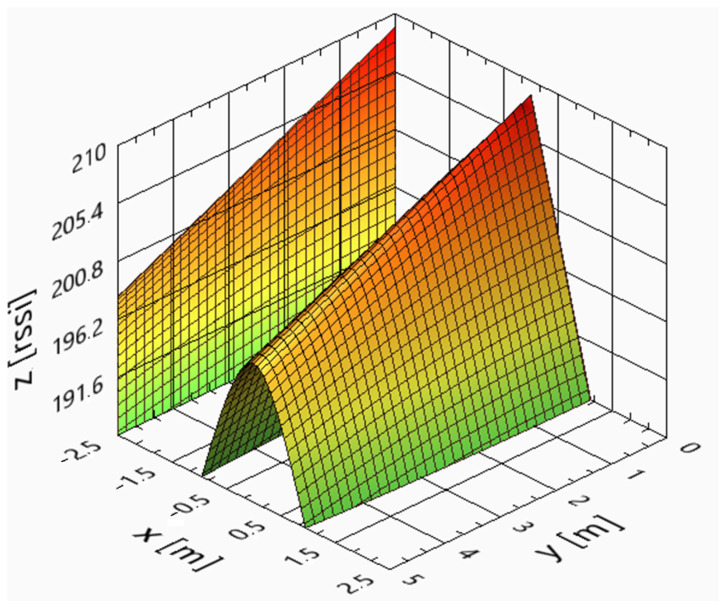
Theoretical RSSI [dBm] surface of the CF-RA1202 antenna when the area is 5 × 5 m.

Using (4) and a 0.5 m step size, the resulting real and theoretical curves can be seen in Figure 16 and Figure 17, respectively, for a CF-RA1202 antenna.

Figure 16 and Figure 17 were computed using the values of Table 3, showing that the theoretical surface envelope of RSSI is very close to the real envelope.

The mathematical surface of the antenna’s RSSI is utilized to estimate the location of the passive tags when two identical antennas are positioned side by side in front of the tags, as illustrated in Figure 15.

### 5.2. Intersection Curves for Tag Position

**Figure 18:**
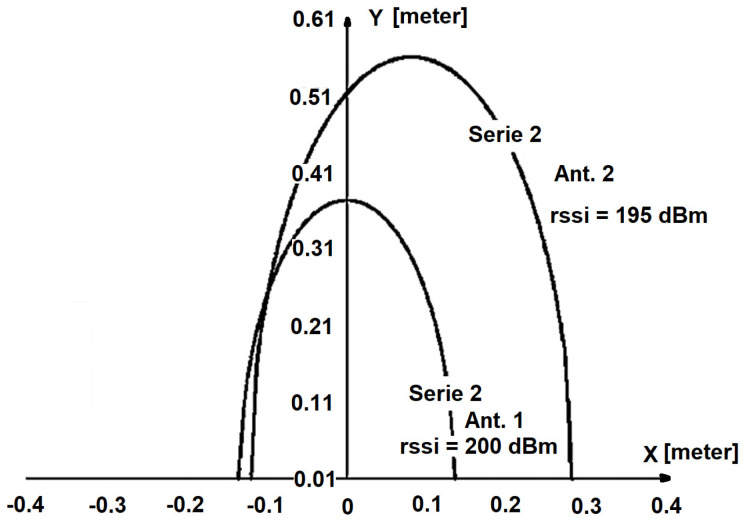
RFID intersection curves.

To determine the position of the tags, a model was employed to calculate the intersection of the antenna curves when placed side by side, using (4). The first antenna was situated at coordinate (0, 0), with an RSSI value of *z*_1_ (as shown in Figure 18), and the second antenna was positioned at coordinate (*x*_0_, 0), with an RSSI value of *z*_2_ [47].

(5) $$y=\frac{L}{\alpha -\beta }\sqrt{{\left(\alpha -z_{1}\right)}^{2}-{{\gamma_{1}}^{2}x}^{2}}.$$

In Figure 18 the RSSI curve of antenna 1, located at (0, 0), with an RSSI value of *z*_1_ = 200, is represented as “Serie 1”, whereas the RSSI curve of antenna 2, located at (0, 0.08 m), with an RSSI value of *z*_2_ = 200, is represented as “Serie 2”. In this specific experiment, the tag is located at (−0.10, 0.25) in meters.

The second antenna is located at (*x*_0_, 0) and has an RSSI value of *z*_2_ (6), which can be expressed as follows:

(6) $$y=\frac{L}{\alpha -\beta }\sqrt{{\left(\alpha -z_{2}\right)}^{2}-{\gamma^{2}\left(x-x_{0}\right)}^{2}}.$$

The *x* coordinate intersection of these two curves ((5) and (6)) is as follows:

(7) $$x=\frac{z_{1}\left(z_{1}-2\alpha \right)+z_{2}\left(2\alpha -z_{2}\right)+\gamma^{2}x^{2}}{2\gamma^{2}x_{0}}.$$

To obtain the *y*-coordinate of the intersection between the two antennas, we substitute (7) into either (5) or (6).

### 5.3. Model Results

Table 4 shows the results obtained when the mathematical model was applied using real values. In this example, it is assumed that the antennas are placed side by side, with a distance of 50 centimeters between them.

Table 4 presents the results of the model tested with five different tags placed at various positions in the (*x*, *y*) space. The RSSI values of each tag, recorded at the antennas’ locations, were used to calculate their positions, resulting in the computed coordinates (*x′*, *y′*). The error was determined by comparing the actual position with the estimated position. In this test, conducted under ideal conditions, the model achieved an average error of 0.5 m. While these tests were not conducted in real-world environments, the results are promising, with a relatively small 50 cm error. The RFID indoor positioning system, based on preliminary testing, utilizes short-range antennas and passive tags with a range of up to 5 m, and the model was initially tested using two static antennas without rotation.

### 5.4. Positioning Error Calculation

The Euclidean positioning error is given by

(8) $$E=\sqrt{{\left(x^{\prime }-x_{real}\right)}^{2}+{\left(y^{\prime }-{xy}_{real}\right)}^{2}}.$$

where

- *x*′ and *y*′ are the estimated coordinates from the model.
- *x_real_* and *y_real_* are the actual coordinates of the tag.

Using the Equation (7):

(9) $$x^{\prime }=\frac{z_{1}\left(z_{1}-2\alpha \right)+z_{2}\left(2\alpha -z_{2}\right)+\gamma^{2}x^{2}}{2\gamma^{2}x_{0}}.$$

and from Equation (5) or (6):

(10) $$y^{\prime }=\frac{L}{\alpha -\beta }\sqrt{{\left(\alpha -z_{2}\right)}^{2}-{\gamma^{2}\left(x^{\prime }-x_{0}\right)}^{2}}.$$

Thus, the positioning error in terms of RSSI values is as follows:

(11) $$E_{x}=\frac{z_{1}\left(z_{1}-2\alpha \right)+z_{2}\left(2\alpha -z_{2}\right)+\gamma^{2}x^{2}}{2\gamma^{2}x_{0}}-x_{real}.$$

(12) $$E_{y}=\frac{L}{\alpha -\beta }\sqrt{{\left(\alpha -z_{2}\right)}^{2}-{\gamma^{2}\left(x^{\prime }-x_{0}\right)}^{2}}-y_{real}.$$

Finally, the total error is as follows:

(13) $$E=\sqrt{{E_{x}}^{2}+{E_{y}}^{2}}$$

### 5.5. Accuracy Estimation

To measure the accuracy of the model, when the variance of the dataset is equal to 1, we define the Mean Squared Error (MSE) and its inverse:

(14) $$MSE=\frac{1}{N}\sum_{i=1}^{N}E_{i}^{2},$$

(15) Accuracy=1−MSE.

where *N* is the number of tested tags and *E_i_* is the error for each tag.

Computing this over the values in Table 4:

(16) $$MSE=\frac{\left(0.3^{2}+{\;0.2}^{2}+{\;1.0}^{2}+{\;0.2}^{2}+{\;0.6}^{2}\right)}{5}=0.338$$

Then, the *Accuracy* is 66.2%.

### 5.6. Error Propagation (Uncertainty Estimation)

If the RSSI values fluctuate due to noise (*σ_z_*), the uncertainty in the estimated coordinates can be computed using partial derivatives.

Uncertainty in *x*′:

(17) $$\sigma_{x}=\left|\frac{\delta x^{\prime }}{\delta z_{1}}\right|\sigma_{z}+\left|\frac{\delta x^{\prime }}{\delta z_{2}}\right|\sigma_{z}$$

Computing the derivatives:

(18) $$\frac{\delta x^{\prime }}{\delta z_{1}}=\frac{2z_{1-2\alpha }}{2\gamma^{2}x_{0}},\;\frac{\delta x^{\prime }}{\delta z_{2}}=\frac{2\alpha -2z_{2}}{2\gamma^{2}x_{0}}$$

(19) $$\sigma_{x}=\left(\frac{\left|z_{1-2}\right|}{2\gamma^{2}x_{0}}+\frac{\left|\alpha -z_{2}\right|}{2\gamma^{2}x_{0}}\right)\sigma_{z}$$

Uncertainty in *y*′:

(20) $$\sigma_{y}=\left|\frac{\delta y^{\prime }}{\delta z_{1}}\right|\sigma_{z}+\left|\frac{\delta y^{\prime }}{\delta z_{2}}\right|\sigma_{z}$$

(21) $$\frac{\delta y^{\prime }}{\delta z_{1}}=\frac{-L\left(\alpha -z_{1}\right)}{\left(\alpha -\beta \right)\sqrt{{\left(\alpha -z_{1}\right)}^{2}-{\gamma^{2}\left(x^{\prime }-x_{0}\right)}^{2}}}$$

(22) $$\sigma_{y}=\frac{-L\left|\alpha -z_{1}\right|}{\left(\alpha -\beta \right)\sqrt{{\left(\alpha -z_{1}\right)}^{2}-{\gamma^{2}\left(x^{\prime }-x_{0}\right)}^{2}}}\sigma_{z}$$

Thus, the total positioning uncertainty is as follows:

(23) $$\sigma_{E}=\sqrt{{\sigma_{x}}^{2}+{\sigma_{y}}^{2}}$$

The uncertainty is computed using error propagation, showing how fluctuations in RSSI affect position estimates.

## 6. IPS Applied to a “Smart” Wheelchair

Our goal was to address the challenge faced by blind individuals who require a wheelchair by developing an “intelligent” wheelchair using recycled materials and low-cost electronics. To enable autonomous navigation, we designed and implemented a long-range RFID localization system as an alternative to the traditional GPS, specifically for indoor environments. Our RFID-based system operates seamlessly inside buildings over extensive areas.

The potential applications of our RFID tracking system with long-range antennas extend far beyond wheelchair users. It could be utilized to track or locate any object or individual within a large area of a building. For example, by attaching passive RFID tags to their personal belongings, a blind person could easily locate thousands of items scattered across their home or workplace. Additionally, the system can assist visually impaired individuals in navigating public spaces by locating strategically placed RFID tags. Furthermore, it can facilitate the retrieval of shared objects in uncertain locations, allowing blind individuals to locate them with ease using our long-range RFID technology.

**Figure 19:**
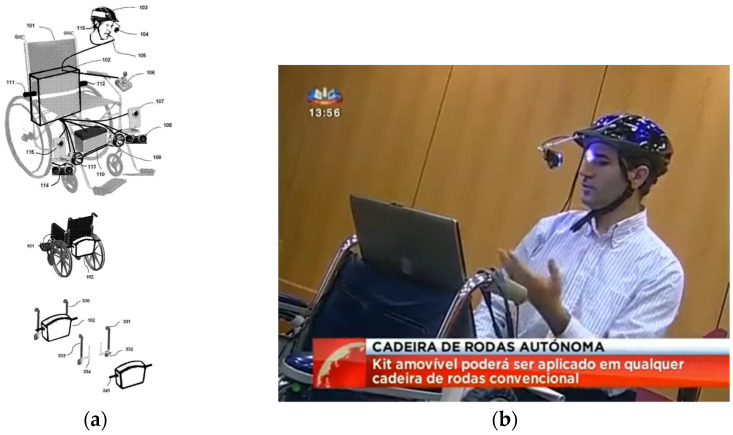
(**a**) Diagram of the “smart” wheelchair from utility model no. 11027. (**b**) “Smart” wheelchair controlled by voice and eye movements using a helmet with a webcam and microphone.

In 2014, the National Institute of Industrial Property (INPI) granted a utility model (No. 11027) titled “Mechanical and Electronic Device for Wheelchairs” to the researcher João Pereira [48]. Figure 19a illustrates the original drawingof the “intelligent” wheelchair developed based on this utility model. The wheelchair kit features a motorization system and a control mechanism that enables voice and eye-movement control through a helmet equipped with a webcam and microphone (Figure 19b).

**Figure 20:**
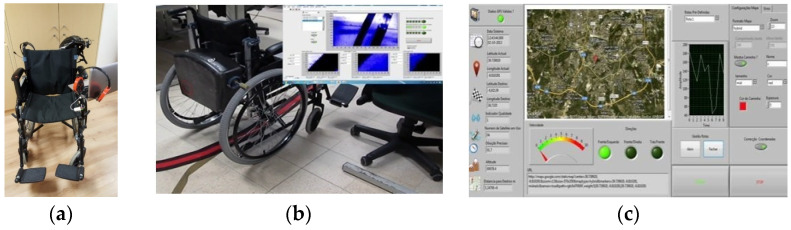
Current version of the “smart” wheelchair: (**a**) With computer support and three-dimensional sound headphones. (**b**) With a colored tracking line system on the floor. (**c**) Wheelchair localization on Google Maps.

Over the past few years, we have focused on improving the initial prototype by integrating new features into the latest model of the “smart” wheelchair. The current version, as shown in Figure 20a,b, replaces the original starter kit with a carbon fiber suitcase developed by the Mechanical Engineering Department at the Polytechnic University of Leiria. We also incorporated a computer and developed a custom headset designed for three-dimensional audio.

To further enhance the autonomous navigation of the “intelligent” wheelchair, we implemented a solution for tracking a colored line on the floor. The line, as shown in Figure 20b, guides the wheelchair along a predefined path. Additionally, to support autonomous driving outdoors, we developed an application that uses GPS coordinates to track the wheelchair’s location on Google Maps. Figure 20c shows the graphical interface of our GPS tracking application.

Recently, we have developed an innovative long-range RFID system that enables the tracking of objects or individuals in indoor environments where the GPS is not feasible. This system extends beyond wheelchair tracking and can be used to locate any object equipped with a passive RFID tag. Simply attaching a low-cost RFID tag to an object is all that is needed to track it. Our advanced RFID tracking system is capable of pinpointing people or objects over a broad area, with passive RFID tags readable at distances of up to 15 m. This project earned the INOVA+ Prize in the “Scientific Excellence” category for digital solutions aimed at building resilient cities (https://premio.inova.business/edicao-2022/ accessed on 14 April 2025).

## 7. Experimental Results and Discussion

This section presents a detailed performance evaluation of the proposed RFID-based IPS, its analyzing localization accuracy, error rates, and computational efficiency. The effectiveness of the RSSI-based model is compared with AoA-enhanced positioning.

The experimental validation consisted of the following:

- A controlled indoor test area (5 × 5 m and 10 × 10 m), where passive RFID tags were placed at predefined positions.
- Two system configurations:
  - A baseline RSSI-only method using static antennas.
  - An enhanced AoA-based method using three rotating directional antennas.
- Performance metrics evaluated:
  - Localization accuracy (average error distance).
  - System responsiveness (time to compute a position).
  - Impact of multipath interference and signal attenuation.

The results demonstrate that the AoA-based approach significantly improves the localization accuracy, reducing the average error from 1.0 m (RSSI-only) to 0.49 m in a 10 × 10 m environment.

Our IPS is an integration of RFID readers, antennas, and microcontrollers. An ESP8266 microcontroller transmits tag data via Wi-Fi to a cloud-based server for processing, where localization estimates are computed using the RSSI or AoA-based model and displayed on a web dashboard.

In the first experimental scenario, the maximum range between the RFID readers and the passive RFID tag was 5 m. The two antennas were placed 0.5 m apart. For the initial test, only two fixed RFID readers were used. When relying solely on two RSSI values, the algorithm was able to estimate the position within 1 s.

**Figure 21:**
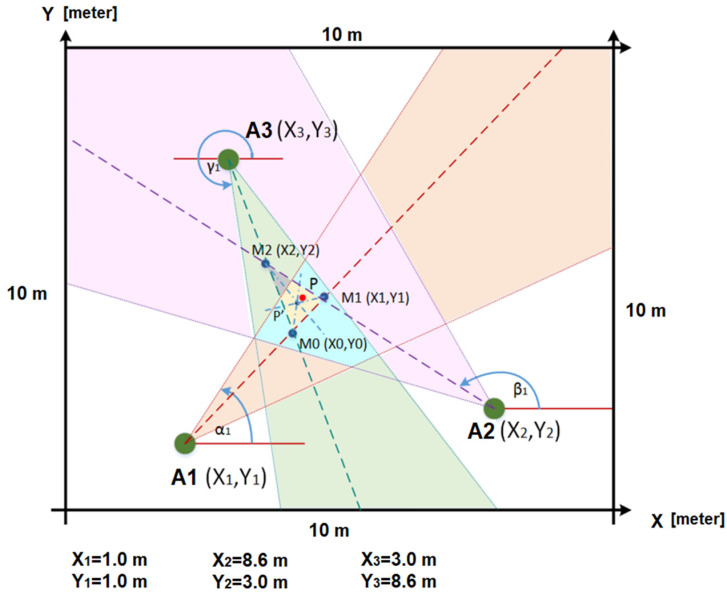
Experimental AoA setup with three directional antennas A1, A2, and A3 [49], inside a square zone of 10 × 10 m.

To enhance the accuracy, later, a new AoA scenario for a 10 × 10 m area was developed using three directional dynamic antennas (A1, A2, and A3 in Figure 21) to track the wheelchair. The rotating antennas used were of two types: Wi-Fi (2.4 GHz) and RFID (867 MHz). The red point in Figure 21 is close to the first intersection M1 (X1,Y1) of the two lines used to estimate the red point by the AoA method, when three rotating antennas (with the same frequency) were placed at the locations A1 (X1,Y1), A2 (X2,Y2), and A3 (X3,Y3). The mathematical formulae used for performing the triangulation can be found in our previous work [49]. We hope to achieve better accuracy from passive RFID tags for our upcoming AoA-based IPS, with more than three rotating antennas.

**Figure 22:**
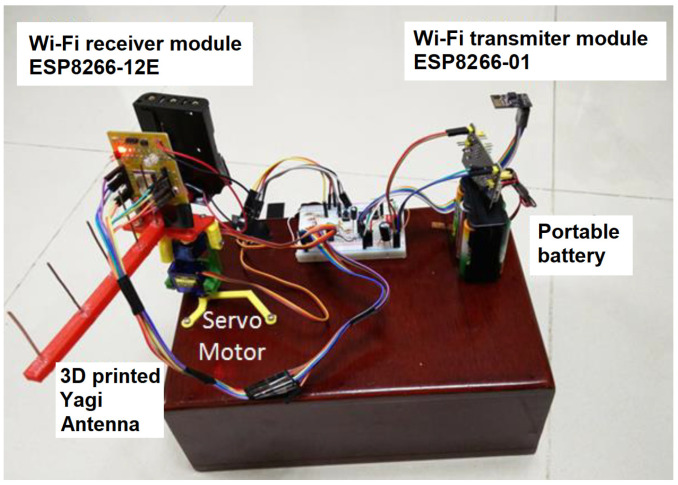
A 3D-printed Yagi antenna to implement our Wi-Fi AoA method [49].

**Figure 23:**
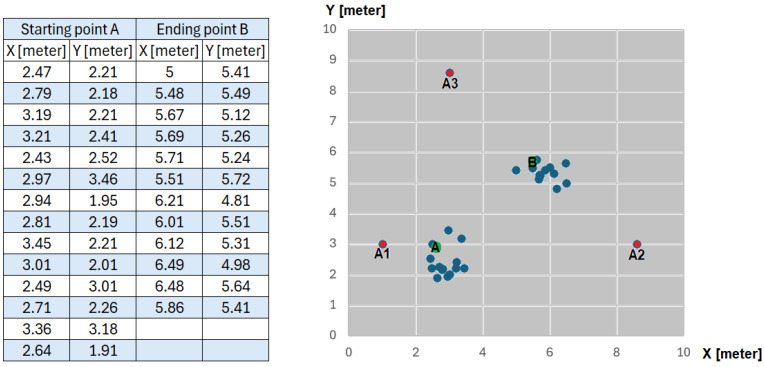
IPS for a 10 × 10 m scenario locating a Wi-Fi module, starting at point A (*X* = 2.6 m, *Y* = 2.9 m) and ending at point B (*X* = 5.6 m, *Y* = 5.75 m) [49], using 3 antennas A1, A2, and A3.

**Figure 24:**
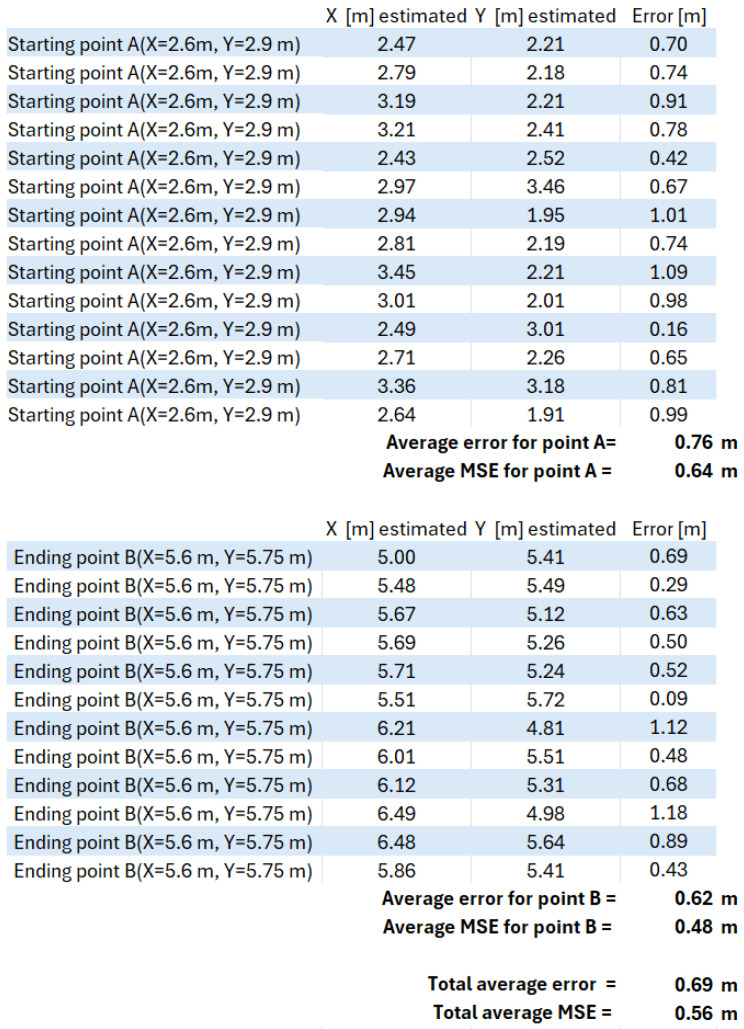
Localization error for our IPS Wi-Fi, with the same 10 × 10 m scenario using a Wi-Fi module, starting at point A and ending at point B [49].

The 10 × 10 m scenario in Figure 21, with the three antennas shown in Figure 22, was implemented in our previous work [49], where the wheelchair localization was performed through a Wi-Fi module. The Wi-Fi module was stationary at point A of Figure 23 for 10 min, and then it was slowly moved to point B, where it remained for 10 min. Figure 23 shows the estimated coordinates for the localization of the Wi-Fi module (or wheelchair), at two points (A and B). The table in Figure 23 has 14 coordinates in position A and 12 coordinates in position B. The localization error for our Wi-Fi IPS with the AoA method was 0.69 m (MSE = 0.56 m), and the values obtained for points A and B are shown again in Figure 24.

**Figure 25:**
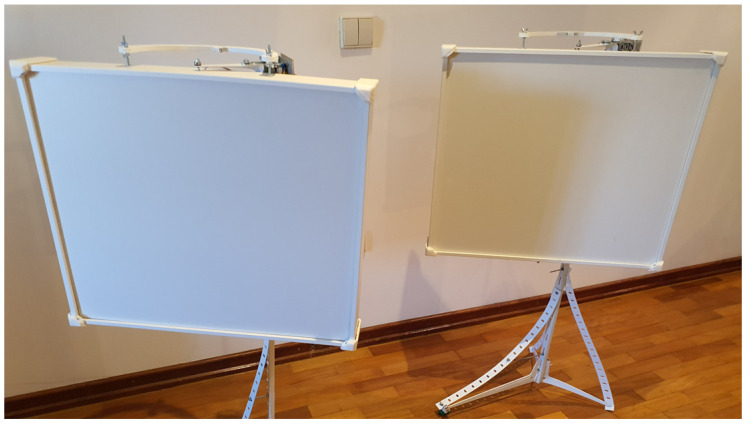
New AoA antennas for RFID (with patent pending).

**Figure 26:**
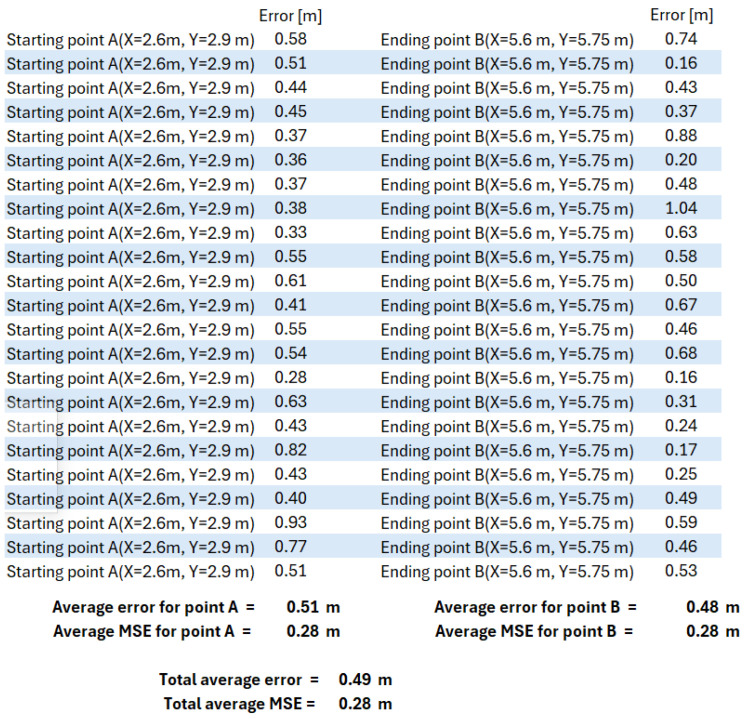
Localization error for our IPS RFID method with AoA, with the same 10 × 10 m scenario using an average localization of 30 passive RFID tags, when the wheelchair starts at point A and ends at point B [49].

The same previous scenario was reused, replacing the Wi-Fi antennas with three rotating RFID antennas, as shown in Figure 25. The “Total average error” value in Figure 26 shows that this error was 0.49 m, with an MSE of 0.28 m. The new RFID IPS prototype of this rotating AoA system had limitations, as it required 30 s to complete a full 180° rotation, performing almost 50 RFID tag readings per second, with a rotational accuracy of 4 degrees.

This new RFID AoA-based approach is still under development, with ongoing optimizations to improve its real-time tracking performance. The initial prototype can be viewed at https://deep-ai-plus.com (accessed on 14 April 2025).

In summary, and using the method described in Section 5, for an RFID-based localization system operating in a 10 × 10 m test area (Figure 21), the RSSI-based method resulted in a localization error exceeding 1 m, using only three antennas (“RSSI-based with passive RFID tags” in Table 5). In contrast, the RFID AoA-based method significantly improved the accuracy, achieving an average localization error of only 0.49 m for the same tracking distance. The same IPS tests inside an area of 10 × 10 m were implemented with a GPS replacing the RFID passive tag. All of the results of the mean values of the localization errors are presented in Table 5.

Some error precision values are shown in Table 5. The AoA approach significantly reduced localization errors, achieving an average accuracy improvement of 50% over the initial RSSI-based model.

Currently, existing systems that locate multiple objects in a given area can be prohibitively expensive. Our goal is to offer an affordable and reliable RFID tracking [50] solution that is capable of accurately reading passive RFID tags over large areas, even in environments with thousands of tagged objects. The price of a passive RFID tag is considerably lower than that of a Wi-Fi module, and it can achieve an IPS error accuracy of 0.49 m (as seen in Table 5). The potential applications of this low-cost RFID tracking system are diverse, ranging from hospitals and residential settings to industries and commercial spaces, where many rotating RFID antennas can be spread throughout the internal and external enclosure [51].

## 8. Conclusions

Our RFID IPS demonstrates significant potential for applications in environments such as the Industrial Internet of Things, where precise and scalable object tracking is critical. The system’s performance and accuracy have been validated through preliminary tests, and it is poised for further testing and refinement in an industrial setting.

Looking ahead, we aim to enhance this system by integrating a new long-range antenna utilizing the AoA localization method. This will extend the operational range to 5–30 m, further enhancing the system’s versatility and enabling more comprehensive tracking across larger areas. The potential to deploy this technology in real-time industrial applications opens up opportunities for improving supply chain management, inventory control, and asset tracking, making it a valuable tool for industries adopting IIoT solutions.

In addition, we plan to explore further improvements in accuracy, signal processing algorithms, and system scalability to meet the growing demands of diverse industrial sectors. By refining the system’s capabilities, we envision its broad application in smart factories, warehouses, logistics, and beyond, contributing to the realization of fully connected, intelligent industrial ecosystems.

## Figures and Tables

**Table 1:** Differences between several RFID frequencies.

Band + Frequency	Read Range	Advantages	Application
Low Frequency (LF) 30–300 KHz	Up to ~50 cm	+ Good penetration in moist environments + No Anti-collision - Slow data rate	Animal tracking Access control Vehicle locks
High Frequency (HF) 3–30 MHz	Up to 1 m	+ Good penetration in moist environments - Poor performance in metal environments	Item tagging Libraries Smart cards Airline baggage
Ultra-High Frequency (UHF) 300–3000 MHz	Passive up to ~25 m Active up to ~100 m	+ Fast data rates + Good performance in metal environments	Supply chain uses Baggage handling Toll collection
Super-High Frequency (SHF) 3–30 GHz	Up to 2 m	+ Fast data rates + Good performance in metal environments - Poor performance in moist environments - High cost	Item tracking Toll collection

**Table 2:** Variables of (3).

Symbol	Description
*x*	Cartesian coordinate *x* of the tag, in meters.
*y*	Cartesian coordinate *y* of the tag, in meters.
*z*	RSSI value obtained by the RFID reader.
*α*	$\mathrm{Maximum}\;\mathrm{RSSI}\;\mathrm{value}\;\mathrm{at}\;\mathrm{the}\;\mathrm{coordinate}\;(x_{0}$, 0).
*β*	$\mathrm{RSSI}\;\mathrm{value}\;\mathrm{at}\;\mathrm{the}\;\mathrm{coordinate}\;(x_{0}$, L).
*L*	Maximum range of the RFID antenna.
γ	Integer value depending on the type of the antenna used (short- and long-range). In this case, the value is 20 for a long-range antenna.
$x_{0}$	Shift value of the antenna distance over the *x* axis.

**Table 3:** Graphic.

Symbol	Description
α	209 RSSI (max RSSI measure)
β	198 RSSI (min RSSI measure)
*L*	5 m
γ	20
$x_{0}$	0 m and 0.5 m

**Table 4:** RFID IPS error results for a 5 × 5 m RSSI-based scenario.

Tag	Ant. 1 at (0, 0) RSSI	Ant. 2 at (0.5, 0) RSSI	*x*′ [m]	*y*′ [m]	*x* Real [m]	*y* Real [m]	Error [m]
1	201	202	0.3	2.5	0.0	2.5	0.3
2	198	196	0.1	4.9	0.0	5.0	0.2
3	194	199	0.6	4.5	0.5	3.5	1.0
4	197	204	0.5	2.2	0.5	2.0	0.2
5	201	195	−0.1	3.6	−0.5	4.0	0.6

Average error = 0.5 m.

**Table 5:** IPS error summary for the 10 × 10m scenario of Figure 21.

Method	Error Precision
GPS receiver (indoor)	1.11 m [49]
RSSI-based with passive RFID tags	1.0 m
AoA-based with one Wi-Fi module	0.69 m [49]
AoA-based with passive RFID tags	0.49 m

## Data Availability

The original contributions presented in this study are included in the article. Further inquiries can be directed to the corresponding author.

## Abbreviations

The following abbreviations are used in this manuscript:

AIDC	Automatic Identification and Data Capture
AoA	Angle of Arrival
CEPT	European Conference of Postal and Telecommunications Administrations
EAS	Electronic Article Surveillance
EPC	Electronic Product Code
ETSI	European Telecommunications Standards Institute
FCC	Federal Communications Commission
FoF	Friend-or-Foe identification
GPS	Global Positioning System
HF	High Frequency, 3 to 30 MHz
IIoT	Industrial Internet of Things
INPI	National Institute of Industrial Property
IPS	Indoor positioning system
ISM	Industrial, Scientific, and Medical bands
ISO	International Standards Organization
kNN	k-Nearest Neighbor
LF	Low Frequency, 30 to 300 kHz
LOS	Line-of-sight
POA	Place of Arrival
RFID	Radio-Frequency Identification
RSS	Received Signal Strength
RSSI	Received Signal Strength Indicator
RSP	Received Signal Phase
RTLS	Real-Time Location System
SKU	Stock-Keeping Unit
TDMA	Time-Division Multiple Access
TDOA	Time Difference of Arrival
TOA	Time of Arrival
UHF	Ultra-High Frequency
USA	United States of America

## References

[1] Tooling4g. https://app.toolingportugal.com/tooling4g-sessao-de-apresentacao-de-resultados/.

[2] Bagarić J., Ferreira M., Pereira J.S., Priem-Mendes S. On estimating indoor location using wireless communication between sensors. Proceedings of the ConfTele 2015.

[3] Ferreira M., Bagarić J., Lanza-Gutierrez J.M., Priem-Mendes S., Pereira J.S., Gomez-Pulido J.A. (2015). On the Use of Perfect Sequences and Genetic Algorithms for Estimating the Indoor Location of Wireless Sensors. Int. J. Distrib. Sens. Netw..

[4] IndoorAtlas. https://www.indooratlas.com/.

[5] Pozyx. http://pozyx.io/.

[6] Nexttome. https://www.nextome.net/en/indoor-positioning-technology.php.

[7] Rcsoft. https://www.rcsoft.pt/.

[8] View. https://www.innovationleader.com/.

[9] Pereira J. (2015). Sequências Perfeitas para Sistemas de Comunicação.

[10] Wu J. (2012). Three-Dimensional Indoor RFID Localization System.

[11] Lazaro A., Girbau D., Villarino R. (2009). Effects of Interferences in Uhf Rfid Systems. Prog. Electromagn. Res..

[12] (2013). Information Technology—Radio Frequency Identification for Item Management—Part 6: Parameters for Air Interface Communications at 860 MHz to 960 MHz.

[13] Pereira J., Bagaric B., Mendes S.P.M. (2016). Standing Wave Cancellation—Wireless Transmitter, Receiver, System and Respective Method. Portuguese Patent.

[14] Pereira J., Gasparovic M., Pujari P., Manjunath G. (2016). Standing Wave Cancellation and Shadow Zone Reducing Wireless Transmitter, System and Respective Method and Uses. Portuguese Patent.

[15] Pereira J., Gasparovic M., Ferreira M.P.M. (2017). Indoor Positioning System and Method. Portuguese Patent.

[16] Lee H.J., Lee M.C. Localization of Mobile Robot Based on Radio Frequency Identification Devices, SICE-ICASE. Proceedings of the International Joint Conference.

[17] Han S.S., Lim H.S., Lee J.M. (2007). An Efficient Localization Scheme for a Differential-Driving Mobile Robot Based on RFID System. IEEE Trans. Ind. Electron..

[18] Bekkali A., Sanson H., Matsumoto M. RFID indoor positioning based on probabilistic RFID map and Kalman filtering. Proceedings of the 2007 International Conference on Wireless and Mobile Communications.

[19] Liu H., Darabi H., Banerjee P., Liu J. (2007). Survey of wireless indoor positioning techniques and systems. IEEE Trans. Syst. Man Cybern. Part C (Appl. Rev.).

[20] Zhou Z., Yang Z., Wu C., Liu Y., Xi W. (2012). Sensorless sensing with RFID. IEEE Trans. Mob. Comput..

[21] Gu Y., Lo A., Niemegeers I. (2009). A survey of indoor positioning systems for wireless personal networks. IEEE Commun. Surv. Tutor..

[22] Zafari F., Gkelias A., Leung K.K. (2019). A survey of indoor localization systems and technologies. IEEE Commun. Surv. Tutor..

[23] CVision. http://www.cvisiontech.com/library/document-automation/data-entry/automatic-data-capture.html.

[24] Bai Y.B., Wu S., Wu H., Zhang K. (2012). Overview of RFID-Based Indoor Positioning Technology.

[25] Arizona’s University. https://sites.arizona.edu/.

[26] What Is EPC RFID Standard?. https://www.cxjrfidfactory.com/what-is-epc-rfid-stardard/.

[27] Bach M.P., Zoroja J., Loupis M. (2016). RFID usage in European enterprises and its relation to competitiveness: Cluster analysis approach. Int. J. Eng. Bus. Manag..

[28] Ecorfid. http://ecorfid.electronicacerler.com/index.php/en/rfid-tag-electronic-product.

[29] Lowry Solutions. https://lowrysolutions.com/blog/a-guide-to-understanding-uhf-passive-rfid-antennas/#:~:text=RFID%20antennas%20perform%20two%20very,data%20from%20multiple%20tags%20simultaneously.

[30] AnalogicTips. https://www.analogictips.com/rfid-tag-and-reader-antennas/.

[31] Tutorial’s Point. https://www.tutorialspoint.com/electronic-product-code-epc#:~:text=Electronic%20Product%20Code%20(EPC)%20is,and%20people%2C%20and%20track%20them.

[32] The Free Dictionary. https://encyclopedia2.thefreedictionary.com/Electronic+Product+Code.

[33] A Look at the Recent Wireless Positioning Techniques with a Focus on Algorithms for Moving Receivers. https://www.researchgate.net/figure/Location-by-ranges-such-as-RSS-or-TOA-measurements_fig1_309846689.

[34] Darcy P., Pupunwiwa P., Stantic B. (2011). The Challenges and Issues Facing the Deployment of RFID Technology. Deploying RFID—Challenges, Solutions, and Open Issues.

[35] Bouet M., Santos A.L.D. RFID Tags: Positioning Principles and Localization Techniques. Proceedings of the Wireless Days, WD ‘08. 1st IFIP.

[36] Environment-Aware Location Estimation in Cellular Networks. https://www.researchgate.net/figure/Location-estimation-via-circle-intersection-using-RSS-values-from-serving-and-neighbor_fig5_220057312.

[37] Chawla K., McFarland C., Robins G., Shope C. Real-Time RFID Localization Using RSS. Proceedings of the International Conference on Localization and Global Navigation Satellite System.

[38] Khan U.H., Rasheed H., Aslam B., Fatima A., Amin L.S.Y., Tenhunen H. (2017). Localization of Compact Circularly Polarized RFID TagUsing ToA Technique. Radioengineering.

[39] Ai Y.L.Z. Research on the TDOA Measurement of Active RFID Real Time Location System. Proceedings of the 2010 3rd International Conference on Computer Science and Information Technology.

[40] A New Geometric Approach to Mobile Position in Wireless LAN Reducing Complex Computations. https://www.researchgate.net/figure/Position-determination-techniques-a-TOA-b-TDOA-c-AOA_fig1_241168823.

[41] Zhanga C., Lib Y. (2011). RFID Localization System Based on K-Nearest Neighbor Algorithm and Extreme Learning Machine Algorithm with Virtual Reference Tags. Comput. Sci. Eng..

[42] Vahedi E., Shah-Mansouri V., Wong V.W., Blake I.F. A Probabilistic Approach for Detecting Blocking Attack in RFID Systems. Proceedings of the Communications (ICC), 2010 IEEE International Conference.

[43] Using RFID Tags for Home Security Monitoring. https://www.embedded.com/using-rfid-tags-for-home-security-monitoring.

[44] Chafon=565. https://www.woah.org/en/what-we-do/standards/codes-and-manuals/terrestrial-manual-online-access/.

[45] Chafon=590. https://www.chafon.com/productinfo/1070103.html.

[46] Chafon’s SDK. https://www.chafon.com/Download.

[47] Pereira J., Gomes H., Mendes S., Santos R., Faria S., Neves C. (2020). Long Range RFID Indoor Positioning System with Passive Tags. Industry 4.0—Shaping the Future of the Digital World.

[48] Pereira J., Martinho M., Martinho P., Silva C. (2014). Utility Model No. 11027 Entitled Mechanical and Electronic Device for Wheelchairs, by INPI (National Institute of Industrial Property). https://pt.espacenet.com/publicationDetails/biblio?II=3&ND=3&adjacent=true&locale=pt_PT&FT=D&date=20140715&CC=PT&NR=11027T&KC=T.

[49] Taipe D. (2017). Indoor and Outdoor Localization System for a Mini Unmanned Autonomous Aerial Vehicle Using Wi-Fi Modules. Master’s Dissertation.

[50] Silva R. (2022). Mobile Long Range rfid Reader for Indoor Positioning System (mlrips). Master’s Thesis.

[51] Wu Y., Lin J., Chen H., Lan H., Yang L. (2025). A transformer-based double-order RFID indoor positioning system. Expert Syst. Appl..

